# Epidemiology and Clinical Course of Haemorrhagic Fever with Renal Syndrome in New Endemic Area for Hantavirus Infection in Croatia

**DOI:** 10.3390/life13081767

**Published:** 2023-08-18

**Authors:** Đurđica Cekinović Grbeša, Nino Zahirović, Viktorija Flego, Marija Livajić, Mari Rončević Filipović, Samira Knežević, Irena Slavuljica

**Affiliations:** 1 Clinic for Infectious Diseases, Clinical Hospital Center Rijeka, 51000 Rijeka, Croatia; viktorija.flego@gmail.com (V.F.); marija.livajic@medri.uniri.hr (M.L.); marirf@medri.uniri.hr (M.R.F.); samira.knezevic.rijeka@gmail.com (S.K.); irena.slavuljica@medri.uniri.hr (I.S.); 2 Department for Infectious Diseases, School of Medicine, University of Rijeka, 51000 Rijeka, Croatia; nino.zahirovic@uniri.hr

**Keywords:** epidemiology, hantavirus, outbreak

## Abstract

Background: Hantaviruses remain an important case of emerging and re-emerging infections in human medicine. This study aimed to analyse the epidemiology, clinical presentation, and outcome of hantavirus infections in the western part of Republic of Croatia, a new geographical area for hantavirus infections. Methods: Retrospective analysis of medical records of patients treated for hemorrhagic fever with renal syndrome (HFRS) at the infectious diseases Clinic of the Clinical Hospital Center in Rijeka, Croatia, from 1 January 2014, to 31 December 2021. Results: During the eight-year period, 251 patients were hospitalized and treated for HFRS, with epidemic outbreaks in years 2014 and 2021. Most patients had a typical clinical course of HFRS and received supportive care. Serological analysis revealed the Puumala Virus (PUUV) as the predominant etiology of the disease. Epidemiological analysis revealed clustering of infections in the region of Gorski Kotar and spread to the area on the Mediterranean coast (Adriatic Sea), which was previously considered an area free from hantavirus infections. Conclusions: The presented results indicate the spread of hantavirus infections in Croatia from the central low-lying parts of the country to the tourist-attractive western area adjacent to the Mediterranean coast, which was previously considered free of hantavirus infections.

## 1. Introduction

Hantavirus infections represent emerging infections of the highest priority in epidemiological surveillance and management. An increasing number of species represent viral reservoirs, and there is a growing number of newly identified hantavirus species [1,2]. Taxonomically, human-pathogenic hantaviruses are members of the *Orthohantavirus* genus. In the following, we refer to ortohantaviruses using the term “hantaviruses”. The main clinical syndromes caused by hantaviruses are hantavirus cardiopulmonary syndrome (HCPS), considered the main hantavirus syndrome of the New World and mainly occurring in the Americas, and Hemorrhagic Fever with Renal Syndrome (HFRS), the main clinical syndrome of hantavirus infection in the Old World and mainly occurring in Europe and Asia. Two hantaviruses, Puumala virus (PUUV) and Dobrava–Belgrade virus, predominate in Europe, while the Seoul virus is frequently described as the pathogen causing urban hantavirus infections [1]. Nephropatia endemica is a frequently used synonym for the mild clinical forms of HFRS caused by PUUV, and is widely spread in Scandinavia, central and western Europe, and the European part of the Russian federation [3]. Persistently infected rodents are reservoirs for all known pathogenic hantaviruses, and are transmitted to humans by aerosols. In vitro trials on human intestinal cells infected with PUUV or intragastric inoculation of the virus in animal models have proposed an additional alimentary route of hantavirus infection [4]. Humans are usually dead-end hosts for hantaviruses. Clinical presentation of hantavirus infection varies from subclinical to severe forms of the disease, depending in part on the particular hantavirus causing the disease. However, despite different clinical presentations, numerous studies show that the exact pathologic mechanisms of the disease lead to capillary hyper-permeability, the main pathological feature of hantavirus infections. Moreover, recent research has shown considerable clinical overlap between HCPS and HFRS [5]. Genetic variation between humans has been suggested to determine the severity of the disease, while in rodents such variation may impact susceptibility to hantavirus infection [6,7]. With no available antiviral treatment and no licenced vaccines for hantaviruses in most of the world, these viruses present a significant public health issue. Therefore, preventive measures and raising public awareness around hantaviruses are currently the only tools for protection from hantaviral disease. Here, we aim to analyse the epidemiological and clinical characteristics of HFRS in patients treated in the Clinical Hospital Center Rijeka, Rijeka, Croatia during an eight-year period in order to determine whether the occurrence of hantaviruses should be considered present in western Croatia, a geographical part of the state that was previously regarded as free of hantavirus.

## 2. Materials and Methods

Medical data on patients hospitalized with HRFS between 1 January 2014 and 31 December 2021 were extrapolated from patients’ medical records. The laboratory parameters analysed to assess disease severity were: leukocyte counts and C reactive protein (CRP) as inflammatory markers, lowest platelet counts to estimate the extent of vascular damage, highest creatinine values and 24 h urine output as markers of kidney injury, and highest values of alanine aminotransferase (ALT) to assess the severity of hepatic impairment.

Puumala virus-specific and Dobrava virus-specific IgM/IgG antibodies were analysed in the sera of hospitalized patients with suspected hantavirus infection using an indirect immunofluorescence assay (IFA) (Progen Biotechnik GmbH, Germany) according to the manufacturer’s instructions.

The study was approved by the Ethical Committee of the Clinical Hospital Center Rijeka, Rijeka, Croatia.

## 3. Results

During the eight-year study period, 251 patients were hospitalized for clinical suspicion of hantavirus infection. Clinical diagnosis was based on the presence of fever >38 °C, thrombocytopenia <150 × 10^9^/L, creatinine >ULN, and positive epidemiological anamnesis for outdoor activities and/or direct or indirect contact with rodents.

Among patients with serologically confirmed HFRS, 157 were male and 41 were female (79% vs. 21%). Age distribution varied from 15 to 87 years, with a mean age of 45.74 years; however, a significant number of patients (78 in number) were 50 years and older.

The annual distribution of hospitalized patients presented epidemiological clustering of cases in the year 2014 (42 cases) and year 2021 (134 cases), while in the rest of that period hantavirus infections were sporadic (in each of 2016, 2018, and 2020 there was one reported patient, while there were seven patients in 2017 and twelve patients in 2019).

**Figure 1:**
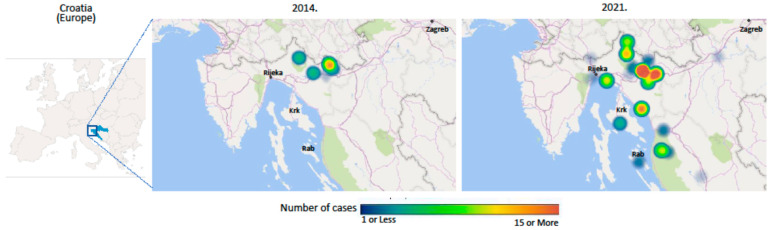
Clustering of hantavirus infections cases in the Gorski Kotar region and north Adriatic coast area in the HFRS outbreak of 2014 and 2021.

Epidemiological analysis revealed the progressive spread of cases of hantavirus infection from previously registered endemic areas in Croatia [8] to the Gorski Kotar region and suburban areas of the city of Rijeka on the Adriatic coast. In 2014, only one patient had a history of visiting previously known endemic hantavirus areas in Croatia, specifically, the Plitvice Lakes National Park in the County of Lika in the central part of Croatia; all other patients were either inhabitants of or visitors to the Gorski Kotar region in the western, highland part of the state (part of Primorsko-Goranska County, one of two western Croatian Counties which were previously considered hantavirus-free areas). In the years 2015–2020, seven patients had a history of living in or visiting the County of Lika, ten patients were inhabitants of or visitors to the Gorski Kotar area, and four patients were inhabitants of towns on the Adriatic coast or the Adriatic island of Rab, areas with no previous history of hantavirus infection. Finally, during the HFRS outbreak in 2021, only seven patients acquired hantavirus infections during occupational work in the County of Lika; 102 of the hospitalized patients were either inhabitants of (94 patients) or visitors to (8 patients) the Gorski Kotar area, 24 patients were inhabitants of either suburban areas of Rijeka, the biggest Croatian port city, Istarska County, the most westerly part of Croatia, or the Adriatic island of Krk, all areas that had never previously reported cases of hantavirus infection [8]. In contrast to previous reports, these data identify the Gorski Kotar region as a new endemic area for hantavirus infection within the Republic of Croatia, in addition to the previously identified areas (see Figure 1) [8].

The majority of patients sought medical attention on the fourth or fifth day from the onset of disease (range 2–21 days, with average hospital admittance 4.84 days from disease onset). The most frequently reported symptoms were fever (189 patients in total), headache (109 patients in total), and lumbar pain (92 patients in total). Additional symptoms included photophobia, blurred vision, nausea and vomiting, arthralgia (especially in the knees), and myalgia.

Blood analysis results revealed a moderate increase in leukocyte count; 57 patients had leukocyte levels above the reference range of 4–10 × 10^9^/L, with the highest value being 17.5 × 10^9^/L, while the remaining patients had leukocyte counts within the reference range. Leukopenia was detected in twelve patients, with the lowest leukocyte count being 2.4 × 10^9^/L. Except for two patients with normal values of C-reactive protein (CRP), all patients had increased levels of CRP, with the highest measured value being 246,8 U/L, the average value 89.3 mg/L, and the reference value 0–5 mg/L. Platelet count analysis revealed 123 patients with significant thrombocytopenia (platelet count less than 100 × 10^9^/L), with the lowest value being 6 × 10^9^/L for a reference value of 158–424 × 10^9^/L. Creatinine analysis showed that 158 patients had values significantly above the top reference value of 90 mmol/L. The highest measured value was 804 mmol/L, with an average of 176.2 umol/L, indicating severe kidney injury in patients hospitalized for HFRS. Notably, only three patients had a history of renal impairment according to previous diseases anamnesis. We analysed 24 h diuresis in patients as an additional marker of kidney injury. Although the average value of 24 h diuresis was normal, at the day of admittance fourteen patients had oliguria and thirteen more developed oliguria during their hospital stay, with the lowest daily diuresis value being 100 mL/24 h. Nine patients entered the polyuric phase of the disease, with the highest daily diuresis value being 4100 mL/24 h. None of the patients had significantly elevated potassium levels that required specific treatment measures.

Hepatic impairment was detected in sixty patients. Among these, the highest measured alanine aminotraspherase (ALT) value was 428 U/L, while the average value was 64 U/L (reference value 5–30 U/L). In addition, we detected increased levels of serum amylases in five patients, with values ranging from 98 to 136 U/L compared to the reference value of 23–91 U/L. These results indicate mild pancreatic involvement, which it is important to point out presented with accompanying abdominal pain in these patients.

During the investigated period, chest X-rays in eleven patients detected pathological findings associated with HFRS. Unilateral lung infiltration was described in six patients, bilateral interstitial infiltration in two patients, and atelectasis in two patients, while bilateral infiltrate with pleural effusion was detected in one patient. The majority (5/9; 55.5%) of these patients were symptomatic, with cough being the most common complaint.

The serological analysis confirmed hantavirus infection in 198 patients (79%), among whom 192 were seropositive for Puumala virus and two patients for Dobrava virus, while four patients had positive IgM antibodies towards both Puumala and Dobrava hantaviruses. Three patients were seronegative for the tested hantaviruses. In 49 patients (21%), serological analysis was not performed due to transient lack of reagents for IFA in the laboratory performing the test. Notably, all of these patients were from the 2021-year HFRS outbreak, during which the increased number of requests for serology testing quickly used up the available reagents. Notably, these patients fulfilled all the clinical and epidemiological criteria (fever >38 °C, thrombocytopenia <150 × 10^9^/L, creatinine > ULN, and positive epidemiological anamnesis for outdoor activities and/or direct or indirect contact with rodents). Despite this, only patients with serological confirmation of hantavirus infection were included in the clinical and disease outcome analyses (Table 1).

In this study, we did not perform serological analysis of the convalescent sera, nor did we analyse viremia.

Treatment included resuscitation of fluids with electrolyte resuscitation and close survey monitoring of vital signs (blood pressure, heart action, and respiration), renal function, and coagulation status. Three patients required hemodialysis. Two patients received a thrombocyte transfusion. Antivirals were not administered to hospitalized patients. Three patients succumbed to infection, all within the first 24 h of hospital admission with clinical presentation of septic shock and multi-organ failure. They were of young age (mean 36 years; range 28 to 42 years) with no underlying medical condition and were serologically confirmed to have PUUV infection. Two were smokers. Interestingly, all of them reported extensive agricultural activities prior to disease onset (hoeing crops); thus, it can be hypothesised that they were potentially infected with higher infective doses of the virus.

Due to the small number of patients infected with Dobrava hantavirus in our study, we could not perform a comprehensive comparison of HFRS severity between PUUV and Dobrava infection.

At a one-month follow-up visit, recovered patients revealed recovery with normal thrombocyte counts, creatinine levels, and ALT levels. Recovered patients felt healthy, with no subsequent complaints regarding normal daily function. In-depth analysis of any potential cardiovascular, renal, or hormonal changes as well as long-term follow-up were not within the scope of this study. The demographic data, clinical parameters, and laboratory values analysed in this study are summarized in Table 1.

## 4. Discussion

This retrospective study covered 251 patients hospitalized with clinical diagnosis of HFRS in the Clinic for Infectious Diseases at the Clinical Hospital Center in Rijeka, Croatia. Hantavirus infections emerge as outbreaks of rising magnitude and amplitude, and present emerging zoonotic infectious disease. Their frequency in humans is considered to be associated with reservoir abundance [9]. As a primary reservoir of PUUV hantavirus in Europe, bank voles colonize the zone of primeval European forests, and PUUV infection outbreaks closely resemble the increase in bank vole populations [10,11,12,13,14]. In Croatia, bank voles are captured in the forests of the middle and east part of the country [15,16], not the Adriatic coast or islands [17,18]. The high infection rate of these rodents was shown to be associated with 2012 winter outbreak of HFRS in the central part of Croatia [17]. In our work, we detected another outbreak of HFRS in 2014, with 46 patients hospitalized within a one-year period, of whom only one had a history of travel to part of Croatia with endemic hantaviruses. All other patients were either inhabitants of, or had a history of staying in the Gorski Kotar region. In the subsequent five years (2015–2019), a significant number of patients were hospitalized for HFRS in our clinic with history of staying in or travel to the Gorski Kotar region, indicating that hantavirus now resides as an endemic pathogen in the Gorski Kotar region and is spreading to the Adriatic coast and islands. A recent outbreak of HRFS in 2021 suggests that Gorski Kotar is an endemic area for hantavirus infection and is involved in the spread of hantaviruses to the Adriatic coast.

The increase in bank vole frequency depends on food availability and the temperature of the previous autumn/winter period, and is considered to be associated with PUUV outbreaks [19]. Indeed, in the 2020/2021 winter period average temperatures in the Gorski Kotar region were 1.7–2 °C higher than normal, which could have impacted the number of bank voles in this area [20].

As in other reports, we noticed a seasonal peak of HFRS incidence in the summer months [9] related to patients with specific behaviour that poses risk of acquiring HFRS. Many patients reported extensive outdoor activity posing a risk of contracting infection for professional reasons as well as direct or indirect contact with small feral rodents. Regardless of sex or age, all those affected were occupationally tied to the woodlands or lived nearby. Notably, common occupations reported in patients’ medical histories included forest work, wood collection, railroad work, and construction work; in addition, retired persons reported spending time in the woods or tending to a garden or orchard. At the beginning of the HFRS outbreak in March and April of 2021, most patients were professional forest workers who were involved in afforestation in the Gorski Kotar area. As the outbreak continued, more patients with HFRS began to report activities other than forest work.

The clinical presentation of hantavirus infection in most patients was HFRS; however, in the 2021 outbreak we identified patients with pulmonary infection involvement as well. Although HFRS is considered distinct from HPS described in the New World, there is now increasing evidence that most if not all features of HPS might be encountered in European HFRS cases as well, which runs contrary to the paradigm that PUUV can only induce HFRS [21,22]. Moreover, pulmonary involvement and arterial desaturation often manifest before any renal deterioration can be detected [22]. Therefore, a combination of fever with blurred vision followed by lung involvement resembling community-acquired pneumonia and thrombocytopenia must alert clinicians to a possible hantavirus infection with HPS features, especially in endemic areas.

Inactivated hantavirus vaccines are available in China and South Korea; in the rest of the world there are no licenced vaccines for the prevention of hantavirus infections [23]. Therefore, a therapeutic approach is the main element of successful treatment. The administration of antivirals in HFRS remains a matter of debate. An American–Chinese trial indicated potential efficacy of ribavirin in HFRS [23], showing reduced mortality in patients receiving the drug within the first week of disease onset. However, meta-analyses have shown no significant effect of ribavirin on the course of hantavirus infection [24,25]. Research on animal models using human monoclonal antibodies has revealed decreased mortality. However, achieving adequate antibody levels for an adequate therapeutic period during specific phases of the disease remains a challenge. Recent data highlight endothelial leakage, mediated mainly by bradykinin, and complementary activation to be the key elements of the pathogenesis of hantavirus infection [26,27]. This has led to a new therapeutic approach in which icatibant, a bradykinin receptor antagonist, has been successful in treating severe cases of hantavirus disease. Similarly, eculizumab, a humanized monoclonal antibody which inhibits the terminal complement membrane attack complex, may be beneficial in treatment of severe hantavirus disease [27,28]. Finally, favipravir, an antiviral drug that inhibits the RdRp protein of the influenza virus, has shown effectiveness against *Bunyaviridae* spp., and is considered to have an adjunctive effect to ribavirin in the treatment of HFRS [29]. As expected, the clinical course of HFRS in most of our patients was favourable, correlating with previously published data showing a mild clinical course and low case fatality rates of <1% for PUUV infection [1]. This implies supportive therapy for our patients, including a survey of vital functions. At the one-month follow-up control, our patients did not report major complaints and their creatinine levels were normal. However, past hantavirus infection can be associated with long-term chronic cardiovascular, renal, and endocrine disorders [27,30]. This implies a serious approach to patients with HFRS paired with studious follow-up.

## 5. Conclusions

This retrospective study established that PUUV predominates in the Gorski Kotar region in the western part of Croatia, and is spreading to coastal marine areas with growing involvement in tourist activity and that share a border with Republic of Slovenia. This finding urges the necessity of educating the population about the epidemiological characteristics of hantavirus infection and applying public health measures to prevent further spread of PUUV infection. Additionally, education on possible preventive measures around hantavirus infection remains a public health aim in Croatia.

## Figures and Tables

**Table 1:** Demographic data, clinical presentation, and laboratory parameters in patients with HFRS.

Demographic Data, Clinical Presentation and Laboratory Parameters in Patients with HFRS *			
Category	Values	Median	Ref. Values
sex	male 156 (79%) female 42 (21%)		
age	15–87 years	45.74	
symptoms reported			
duration of symptoms	2–21 days	4.84	
highest body temperature	37.4–40.0 °C	38.07	
lumbar pain	92 (46%)		
headache	109 (55%)		
blurred vision	45 (18%)		
myalgia	78 (39%)		
vomiting	61 (30%)		
laboratory results			
leukocyte count	2.4–17.5 × 10^9^/L	6.84	3.4–9.7 × 10^9^/L
lowest platelets	6–287 × 10^9^/L	89.97	158–424 × 10^9^/L
C-reactive protein	7–246.8 mg/L	89.31	0–5 mg/L
creatinine	44–839 umol/L	176.17	49–90 umol/L
serological testing			
Puumala IgM	192 (97%)		
Dobrava IgM	2 (1%)		
Puumala IgM+ and Dobrava IgM+	4 (2%)		
clinical course and outcome			
mild/moderate clinical course	180 (91%)		
severe renal impairment with hemodialysis request	3 (1.5%)		
hypotension and shock	12 (6%)		
death	3 (1.5%)		

* All parameters and values presented were analysed at the day of admittance.

## Data Availability

Data sharing not applicable.
